# Molecular and Functional Characterization of Neurogenin-2 Induced Human Sensory Neurons

**DOI:** 10.3389/fncel.2020.600895

**Published:** 2020-12-04

**Authors:** Amy J. Hulme, Jeffrey R. McArthur, Simon Maksour, Sara Miellet, Lezanne Ooi, David J. Adams, Rocio K. Finol-Urdaneta, Mirella Dottori

**Affiliations:** ^1^Illawarra Health and Medical Research Institute, Wollongong, NSW, Australia; ^2^School of Medicine, University of Wollongong, Wollongong, NSW, Australia; ^3^School of Chemistry and Molecular Bioscience, University of Wollongong, Wollongong, NSW, Australia; ^4^Molecular Horizons, University of Wollongong, Wollongong, NSW, Australia

**Keywords:** dorsal root ganglia, electrophysiology, excitability, human sensory neurons, ion channels, NGN2, pluripotent stem cells

## Abstract

Sensory perception is fundamental to everyday life, yet understanding of human sensory physiology at the molecular level is hindered due to constraints on tissue availability. Emerging strategies to study and characterize peripheral neuropathies *in vitro* involve the use of human pluripotent stem cells (hPSCs) differentiated into dorsal root ganglion (DRG) sensory neurons. However, neuronal functionality and maturity are limited and underexplored. A recent and promising approach for directing hPSC differentiation towards functionally mature neurons involves the exogenous expression of Neurogenin-2 (NGN2). The optimized protocol described here generates sensory neurons from hPSC-derived neural crest (NC) progenitors through virally induced NGN2 expression. NC cells were derived from hPSCs *via* a small molecule inhibitor approach and enriched for migrating NC cells (66% SOX10+ cells). At the protein and transcript level, the resulting NGN2 induced sensory neurons (_NGN2_iSNs) express sensory neuron markers such as BRN3A (82% BRN3A+ cells), ISLET1 (91% ISLET1+ cells), TRKA, TRKB, and TRKC. Importantly, _NGN2_iSNs repetitively fire action potentials (APs) supported by voltage-gated sodium, potassium, and calcium conductances. In-depth analysis of the molecular basis of _NGN2_iSN excitability revealed functional expression of ion channels associated with the excitability of primary afferent neurons, such as Nav1.7, Nav1.8, Kv1.2, Kv2.1, BK, Cav2.1, Cav2.2, Cav3.2, ASICs and HCN among other ion channels, for which we provide functional and transcriptional evidence. Our characterization of stem cell-derived sensory neurons sheds light on the molecular basis of human sensory physiology and highlights the suitability of using hPSC-derived sensory neurons for modeling human DRG development and their potential in the study of human peripheral neuropathies and drug therapies.

## Introduction

Basic sensory experiences such as pain, temperature, pressure, and spatial positioning require specialized sensory neurons within the peripheral nervous system to detect and transmit stimuli to the central nervous system for processing. Different subtypes of sensory neurons have specific functions in detecting and conveying signals of proprioception (detection of spatial position, movement, muscle pressure, and tension); mechanoreception (cutaneous touch, hair deflection, and vibration), and nociception (pain perception, noxious thermal, mechanical and chemical stimuli). As can be expected with multiple sensory subtypes, there is a plethora of diseases and sensory neuropathies associated with dysfunction in the development or function of each neuronal subtype within the dorsal root ganglia (DRG). These can be caused by, but are not limited to, acquired or inherited/genetic diseases, autoimmune disorders, inflammation, injury, and idiopathic conditions that can result in hypersensitivity, numbness, ataxia, and chronic pain (Johnson et al., 1986; Kuntzer et al., 2004; Sghirlanzoni et al., 2005; Axelrod and Gold-Von Simson, 2007; Damasceno et al., 2008; Martinez et al., 2012). Furthermore, adverse side effects of treatments (e.g., chemotherapeutics for the treatment of cancer) can lead to damage of sensory neurons with peripheral neuropathic symptoms like numbness, chronic pain, and/or tingling. At the cellular level, dysfunction, and damage to sensory neurons can alter the expression, distribution, density, and conductance of membrane ion channels. These changes can dramatically alter action potential (AP) transduction producing changes in the excitability of specific sensory neurons resulting in the symptoms of peripheral neuropathies (Rasband et al., 2001; Chaplan et al., 2003; Chen et al., 2009; Zhang and Dougherty, 2014; Pitake et al., 2019). For example, injury to rodent paws causes an increase in the expression of specific calcium channels in the membrane of sensory neurons resulting in hyper-excitable neurons (Chen et al., 2009). Similarly, chemotherapeutics can alter the expression of ion channels resulting in peripheral neuropathies (Descoeur et al., 2011; Zhang and Dougherty, 2014; Leo et al., 2017). Following application of paclitaxel, a common chemotherapeutic used for several types of cancers, rat DRG neurons manifest altered excitability due to higher expression of voltage-gated potassium, hyperpolarization-activated cyclic nucleotide-gated (HCN), and voltage-gated sodium channels together with a decrease in the expression of inwardly-rectifying potassium channels (Zhang and Dougherty, 2014). Ion channels have a critical role in the development and progression of peripheral neuropathies hence, strides have been made towards the development of cancer therapies aimed at curtailing excitability remodeling and neurotoxicity (Keenan et al., 2020).

Methods to investigate neuronal excitability, ion channel function and dysfunction in sensory neurons have previously relied on animal models. However, inherent species differences limit the translation of rodent-based findings to human neurophysiology and pathology (Zylka et al., 2003; Han et al., 2015; Chang et al., 2018; Rostock et al., 2018; Schwaid et al., 2018). To circumvent the translatability of rodent models and the difficulty in accessing human tissue, the use of human pluripotent stem cell (hPSC) technology has rapidly progressed. These approaches enable human neurons to be derived *in vitro* to investigate all aspects of neuronal function including development, toxicity and diseases while providing a relevant platform for drug screening and delivery.

There are various protocols to derive DRG sensory neurons from hPSCs (Chambers et al., 2012; Blanchard et al., 2015; Boisvert et al., 2015; Schrenk-Siemens et al., 2015; Wainger et al., 2015; Alshawaf et al., 2018; Nickolls et al., 2020). One approach uses small molecule inhibitors to generate a progenitor cell, such as neural crest (NC) cells, which are then transduced to express the transcription factors of interest to drive the differentiation of the NC cells to functional sensory neurons (Schrenk-Siemens et al., 2015; Nickolls et al., 2020). The benefit of this approach is that it mimics neurogenesis and developmental patterning by causing the expression of transcription factors at specific developmental time points. Studies following this method report improved sensory neuron efficiency compared to those that bypass the progenitor states (Schrenk-Siemens et al., 2015; Nickolls et al., 2020). For example, Schrenk-Siemens et al. (2015) and Nickolls et al. (2020) have successfully used small molecules to generate NC cells in which either the expression of Neurogenin-2 (NGN2) or a combination of NGN2 and BRN3A is introduced at the NC stage to mimic the first wave of neurogenesis producing functional sensory neurons (Schrenk-Siemens et al., 2015; Nickolls et al., 2020). Transcriptional analysis of sensory neuron cultures generated with the available protocols has evidenced good efficiency, however, the resulting sensory neuron excitability profiles and functional expression of the underlying conductances have not been thoroughly addressed. Functional characterization of the resulting neuroexcitability profiles is of particular importance, as emerging evidence suggests that the pathophysiology of many sensory neuropathies results from the dysfunction of their electrophysiological properties often arising from a channelopathy. Furthermore, a thorough understanding of the voltage-gated sodium, calcium, and potassium currents underpinning excitability sets the foundation to establish hPSC-derived sensory neuron cultures as platforms for drug screening, drug delivery, and disease modeling. In this study, sensory neurons were derived from hPSCs using a combination of small molecules to generate NC progenitors followed by induced expression of NGN2. This work provides a detailed description of human NGN2 derived sensory neuron’s (_NGN2_iSN) major voltage-gated sodium, calcium and potassium conductances, as well as their functional acid-sensitive currents. The observed excitability profile of _NGN2_iSNs demonstrates the generation of a functionally heterogeneous population of sensory neurons with membrane passive and active electrical properties akin to mature human sensory neurons.

## Materials and Methods

### hPSC Culture

All experiments were approved by the University of Wollongong Human Ethics Committee (#2017-375) and University of Wollongong Institutional Biosafety Committee (#GT19-09). The H9 human stem cell line (WA09, WiCell) was maintained on vitronectin XF™ (#07180, StemCell Technologies) coated T25 flasks using TeSR-E8 (#5990, STEMCELL Technologies), at 37°C 5% CO_2_. When the cultures reached a confluence of 60–70%, they were passaged using 0.5 mM EDTA/PBS^−/−^.

### Lentiviral Production

HEK293T cells were maintained in DMEM/F12 5% Foetal Bovine Serum (FBS; SFBS-F, Interpath) in a T75 flask, passaged using accutase (00-4555-56, Life Technologies), and seeded at a density of 4 × 10^6^ cells/flask, 24 h before transfection. Lentiviral particles were produced in HEK293T cells through co-transfection of the doxycycline-inducible lentiviral vector PLV-TetO-hNGN2-eGFP-PURO (#79823, Addgene) or the reverse tetracycline transactivator vector FUW-M2rtTA (#20342, Addgene) or PLV-TetO-eGFP-PURO (modification of #79823 Addgene vector) with the packaging plasmids vSVG (#8454, Addgene), RSV (#12253, Addgene) and pMDL (#12251, Addgene) using polyethyleneimine (408727, Sigma–Aldrich) and Opti-MEM (#31985062, Life Technologies). The media from the transfected HEK293T cells was changed 6 h after transfection and replaced with DMEM/F12 5% FBS. Viral particles were collected at 24, 48, and 72 h post-transfection, filtered (0.45 μm pore size), and centrifuged at 66,000× *g* for 2.5 h at 4°C. The pelleted viral particles were resuspended in PBS at a 200× enrichment, aliquoted, and stored at −80°C until use. The viral particles were tested on hPSCs to confirm successful production (Supplementary Figure 1).

### hPSC Differentiation to Sensory Neurons

hPSC differentiation to sensory neurons was based on previously published methods by Alshawaf et al. (2018), with modifications. Briefly, hPSC was seeded in an organ culture dish (60 × 15 mm, #353037, Corning) previously coated with 10 μg/ml laminin (#23017015, Thermo Fisher Scientific) for 24 h at 4°C, at a density of 2 × 10^4^ cells/dish in TeSR-E8. Following 24 h (day 1), the media was removed and replaced with neural induction media [Neurobasal medium (#21103-049, Thermo Fisher Scientific), DMEM/F12, 1× N-2 supplement (#17502-048, Thermo Fisher Scientific), 1× B-27 supplement (without vitamin A; #12587-010, Thermo Fisher Scientific), 1× Insulin-transferrin-Selenium-A (51300-044, Thermo Fisher Scientific), 2 mM L-glutamine (25030149, Life Technologies), 0.3% glucose (G8769, Sigma–Aldrich)], supplemented with 3 μM CHIR99021 (SML1046, Sigma–Aldrich) and 10 μM SB431524 (72234, STEMCELL Technologies) and this was repeated on day 3 to direct the differentiation to caudal neural progenitors (CNPs). Neurospheres were formed on day 5 by harvesting the CNPs using 0.5 mM EDTA/PBS^−/−^, centrifuged at 200× *g* for 3 min. CNPs were then resuspended in neuronal media [Neurobasal medium (#21103-049, Thermo Fisher Scientific), 1× N-2 supplement (#17502-048, Thermo Fisher Scientific), 1× B-27 supplement (without vitamin A; #12587-010, Thermo Fisher Scientific), 1× Insulin-transferrin-Selenium-A (#51300-044, Thermo Fisher Scientific), 2 mM L-glutamine (#25030149, Life Technologies)] supplemented with 20 ng/ml FGF2 (#78003, STEMCELL Technologies) and 10 ng/ml BMP2 (RDS355BM010, *in vitro* Technologies) and 100 μl of cell suspension plated into each well of an ultra-low attachment U-bottom 96 well plate (CLS7007, Sigma–Aldrich) and centrifuged at 200× *g* for 4 min. Neurosphere formation was observed following 24 h, and 50 μl/well of neuronal media supplemented with 20 ng/ml FGF2 and 10 ng/ml BMP2 was added on day 8, with full media changes conducted every 3rd day. Before plating the spheres, 12 mm glass coverslips in 24-well plates were coated in 10 μg/ml Poly-D-Lysine (P6407, Sigma–Aldrich) for 30 min at room temperature and then 10 μg/ml laminin at 4°C overnight. To enrich for the migrating NC, on day 12 the neurospheres were plated as whole spheres on the previously coated coverslips in neuronal media supplemented with 10 μM Y-27632 (72302, STEMCELL Technologies). On day 14, the neurospheres were removed using a P200 pipette, leaving behind the migrating NC cells. These cells were then transduced with 2 μl/ml PLV-TetO-hNGN2-eGFP-PURO or PLV-TetO-eGFP-PURO lentiviral particles and 2 μl/ml FUW-M2rtTA lentiviral particles in neuronal media supplemented with 10 μM Y-27632, 10 ng/ml BDNF (78005, STEMCELL Technologies), 10 ng/ml GDNF (78058, STEMCELL Technologies), 10 ng/ml NT-3 (78074, STEMCELL Technologies) and 10 ng/ml ß-NGF (78092, STEMCELL Technologies). To induce NGN2 expression a full media change was conducted on day 15 removing the viral medium and replacing it with neuronal media supplemented with 1 μg/ml doxycycline (D9891, Sigma–Aldrich), 10μM Y-27632, 10 ng/ml BDNF, 10 ng/ml GDNF, 10 ng/ml NT-3 and 10 ng/ml ß-NGF. NGN2 expression was induced by the addition of doxycycline for 96 h (day 15–19). To select for successfully transduced cells 0.5 μg/ml of puromycin (73342, STEMCELL Technologies) was added for 48 h (day 17–19). Media changes were conducted every 2–3 days. On day 22, BrainPhys™ Neuronal Medium (05790, STEMCELL Technologies), 0.02% NeuroCult™ SM1 Without Vitamin A (05731, STEMCELL Technologies), 0.01% N2 Supplement-A (07152, STEMCELL Technologies) was phased into the neuronal media to mature the neurons (25:75, 50:50, 75:25, 100:0 Brainphys: neuronal media every media change) and proliferating cells were removed upon addition of an anti-mitotic agent 2.5 μM cytosine β-D-arabinofuranoside (AraC; C1768, Sigma–Aldrich) between days 25–27 for 48 h. The neurons were matured until day 34 and were then fixed for immunocytochemistry or harvested for RNA. For patch-clamp analysis, the neurons were used between days 34–48.

#### Immunocytochemistry

Cells were washed with PBS three times and fixed with 4% PFA for 20 min at room temperature and then washed three times with PBS. Cells were permeabilized with 0.1% triton/PBS for 10 min and then blocked in 10% donkey serum/PBS (D9663, Sigma–Aldrich) for 1 h at room temperature. Samples were then incubated with the primary antibody in 10% donkey serum/PBS overnight at 4°C. The primary and secondary antibodies were used as per Table 1. Following the overnight incubation, the coverslips were washed with PBS three times for 5 min and then incubated with the appropriate secondary antibody in 10% donkey serum/PBS for 1 h at room temperature. Samples were again washed three times for 5 min in PBS and stained with DAPI (D9542, Sigma–Aldrich) for 15 min and then washed three times for 5 min in PBS. The coverslips were mounted with ProLong™ Gold Antifade Mountant (P36934, Life Technologies Australia) onto microscope slides (MENSF41296P, Thermo Fisher Scientific). Images were taken using a Leica confocal SP8 microscope and were exported and analyzed using ImageJ software.

**Table 1:** Primary and secondary antibody details and dilutions.

Antibody name	The catalog number, company	Dilution
Mouse anti-P75^NTR^	M-1818-100, Biosensis	1:500
Goat anti-SOX10	RDSAF2864, R&D Systems	1:100
Mouse anti-PAX3/7	Sc-365843, Santa Cruz Biotechnology	1:500
Rabbit anti-ISLET1	ab20670, Abcam	1:500
Mouse anti-BRN3A	MAB1585, Millipore	1:500
Mouse anti-PERIPHERIN	MAB1527, Millipore	1:500
Mouse anti-ß- III TUBULIN	MAB1637, Millipore	1:500
Goat anti-TRKA	RDSAF175, R&D Systems	1:400
Mouse anti-TRKB	NOVNBP147898, Novus Biologicals	1:100
Rabbit anti-TRKC	7H3L20, Thermo Fisher Scientific	1:250
Rabbit anti-Nav1.8	ab66743, Abcam	1:200
Rabbit anti-Nav1.7	ab65167, Abcam	1:500
Guinea pig anti-ASIC3	AGP-052, Alomone	1:500
Rabbit anti-PAN-ASIC	ASIC-PAN-51A, Alpha Diagnostic	1:100
Rabbit anti-TRPV1	NB120-3487, Novus Biologicals	1:500
Donkey-anti-mouse IgG-488	ab150109, Abcam	1:500
Donkey-anti-rabbit IgG-555	ab150062, Abcam	1:500
Donkey-anti-goat IgG-647	ab150135, Abcam	1:500
Donkey-anti-mouse IgG-647	ab150111, Abcam	1:500
Goat Anti-Guinea pig IgG H&L-555	ab150186, Abcam	1:500

### RNA Purification, cDNA, and RT-qPCR

RNA was isolated and purified using the PureLink™ RNA Mini Kit (12183025, Thermo Fisher Scientific) kit according to the manufacturer’s instructions. Genomic DNA was removed, and RNA was reverse transcribed into cDNA using the iScript™ gDNA Clear cDNA Synthesis Kit (Biorad, 1725035) in a master cycler. cDNA was diluted to 20 ng/μl and stored at −20°C until used for RT-qPCR. RT-qPCR was conducted using the PowerUP SYBR green master mix (A25778, Thermo Fisher Scientific) and respective primers (Table 2) in a Quantistudio 5, following the manufacturer’s instructions. All targets were internally normalized to three housekeeper genes, *GAPDH, PPIA*, and *B2M*, and expressed as 2^−ΔCt^.

**Table 2:** Primer sequences.

Gene name	Protein name	FW primer 5′-3′	REV primer 5′-3′
GAPDH	GAPDH	TCGGAGTCAACGGATTTGGT	TTCCCGTTCTCAGCCTTGAC
PPIA	PPIA	ACGTGGTATAAAAGGGGCGG	CTGCAAACAGCTCAAAGGAGAC
B2M	β2 microglobulin	AAGGACTGGTCTTTCTATCTC	GATCCCACTTAACTATCTTGG
NTRK1	TRKA	CTCCAACACGGAGGCAATCG	CGCATGATGGCGTAGACCTC
NTRK2	TRKB	TTATGTGGATCAAGACTCTCC	AAACCACAATTGGGTATCTG
NTRK3	TRKC	GATTATTACAGGGTGGGAGG	AAGCTCCATACATCACTCTC
POU4F1	BRN3A	AACTGGACCTCAAAAAGAAC	GATAACGGACACTCCAAATC
ISL1	ISLET1	CTAATATCCAGGGGATGACAG	CTGGTAACTTTGTACTTCCAC
PRPH	PERIPHERIN	GTTCTGATCAAGACCATTGAG	AATTCAGGAGTGGTCTTAGG
SCN9A	Nav1.7	AAAGGGAAAACAATCTTCCG	TGTACTCGACATTTTTGGTC
SCN10A	Nav1.8	AAAGGAGAAGAAGTTCCAGG	GGTTAAAGGTGATCCATTGTG
KCNA2	Kv1.2	AGCCCAGCCCAATCCTAGAG	CTTGGCTGACCAGAGACGTG
CACNA1H	Cav3.2	TCTGGGCTACATCCGGAACC	TCTCCCAGACGCTGATGACC
CACNA1B	Cav2.2	TTGCTTACAAGCGCCTGGTT	CAGCGTGGACGTGAAGTGAA
CACNA1A	Cav2.1	CCTACCGACATGCCCAACAG	ATCTCTGCCCATCTCTCGCA
KCNMA1	KCa1.1	TCACAACAAGGCCCATCTGC	GCCCAACTTCAACTCTGCGA
KCNB1	Kv2.1	AAGATCCTTGCCATAATTTCC	GAACCTCAGCAGGTACTC
HCN1	HCN1	CGAGCTGATACATATTGTCG	CCTATTCGATCTAGTCGGTC
HCN2	HCN2	AGTACCAGGAGAAGTACAAG	TAACGGTGCTCATAGTAGTC
HCN3	HCN3	AAGATGTTCGATGAGGAAAG	GACAGGTGAAGTTAATGATCTC
HCN4	HCN4	GACTTCAGATTTTACTGGGAC	CATCCTTGAAGAAGGTGATG

### Electrophysiology

Whole-cell patch-clamp recordings of _NGN2_iSNs were made at room temperature (20–22°C) with a MultiClamp 700B Amplifier, digitalized with a Digidata 1440 and controlled with pClamp11 software (Molecular Devices, San Jose, CA, USA). Whole-cell membrane currents were sampled at 100 kHz, filtered at 10 kHz, and series resistance compensated 60–80%. Fire-polished borosilicate (1B150F-4, World Precision Instruments, USA) patch pipettes were used with resistance 2–4 MΩ and filled with intracellular solutions containing (in mM): 140 K-Gluconate, 10 NaCl, 2 MgCl_2_, 5 EGTA, and 10 HEPES, pH 7.2. The resting membrane potential (RMP) was recorded immediately after switching into the current-clamp mode as the average membrane voltage in the absence of current injection. Stimulus membrane potentials were determined by injection of increasing bias currents. The rheobase or current threshold was defined as the minimum amount of current necessary to evoke a single AP during 500 ms depolarizing current steps (in 5 pA increments). Elicited APs were counted/plotted as a function of the current injection intensity during stimulation. For voltage-clamp experiments, the extracellular solution was varied depending on the ion channel being examined. For voltage-gated sodium channels, Na^+^ currents (*I_Na_*) were isolated with an extracellular solution containing (in mM): 110 NaCl, 2 CaCl_2_, 2 MgCl_2_, 30 TEA-Cl, 10 D-Glucose, and 10 HEPES, pH 7.3. To isolate Ca^2+^ currents (*I_Ca_*), the extracellular solution contained (in mM): 140 TEA-Cl, 10 mM CaCl_2_, 1 MgCl_2_, 10 HEPES, 10 D-Glucose, pH 7.3. For current-clamp experiments an extracellular solution containing (in mM): 135 NaCl, 2 CaCl_2_, 2 MgCl_2_, 5 KCl, 10 D-Glucose, 10 HEPES, pH 7.3 was used. To examine *K*^+^ currents (*I*_K_) the latter extracellular solution was supplemented with 1 μM TTX. Voltage dependence of activation and steady-state inactivation (SSI) of the various ionic currents were fit by the modified Boltzmann equation:

$$I\;or\;G=1/\left(1+exp\;\left(\frac{Vm-V_{0.5}}{ka}\right)\right)$$

where *I* is the current or *G* is the conductance, *Vm* is the pre-pulse potential, *V*_0.5_ is the half-maximal activation potential and *ka* is the slope factor.

### Pharmacology

Tetrodotoxin citrate was purchased from Abcam (Melbourne, Australia). Kv channel inhibitors were sequentially and cumulatively applied using the most selective antagonists (at concentrations where target selectivity has been verified experimentally) first followed by less selective/potent modulators. Cone snail toxin κ-RIIIJ (B. Olivera, University of Utah) selectively blocks heteromeric Kv1.2 complexes with Kv1.1 or Kv1.6 channels (Cordeiro et al., 2019). Scorpion toxin, Urotoxin (Uro, Alomone Labs, Cat#: STU-200), is a potent inhibitor of homomeric Kv1.2 (Luna-Ramirez et al., 2020). Guangxitoxin-1E (GxTx, Alomone Labs, Cat#: STG-200) was applied (Liu and Bean, 2014) to inhibit Kv2 channels. Subsequent application of 4-aminopyridine (4-AP, Sigma–Aldrich, A78403) was applied to block all Kv3 channels (Gutman et al., 2005) and was followed by AmmTx3 (Smartox Biotechnology, Saint-Egrève, France, AMX001-50010) to inhibit Kv4 channels (Vacher et al., 2002).

## Results

### Induction of NGN2 Expression in hPSC-Derived Neural Crest Cells Generates Sensory Neurons (_NGN2_iSN)

**Figure 1:**
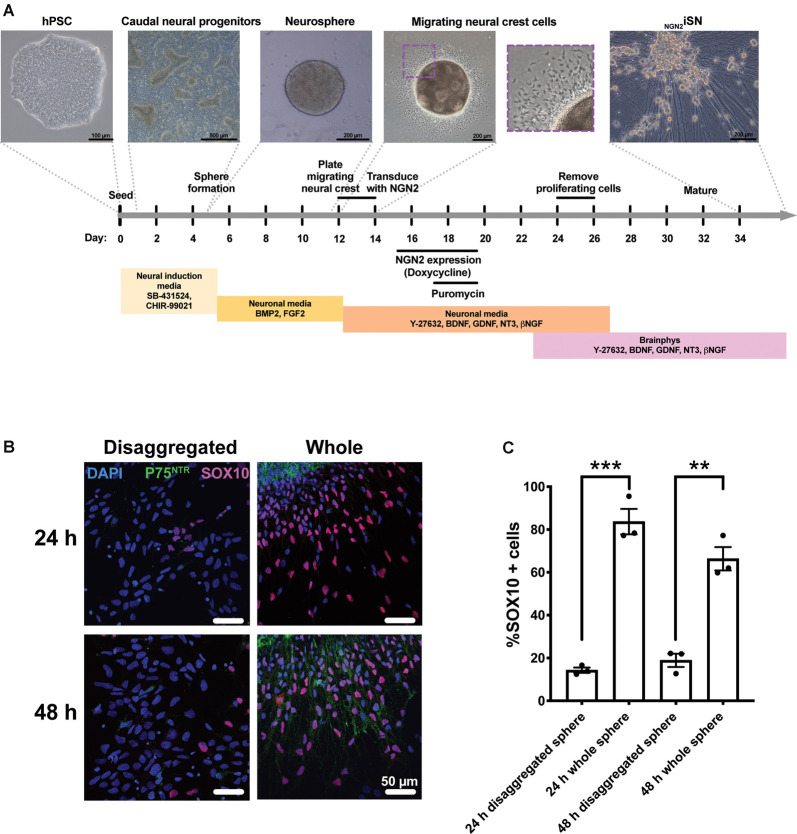
Generation of hPSC-derived Neural crest (NC) cells and _NGN2_iSN.** (A)** Schematic of the protocol to derive _NGN2_iSNs. Briefly, the protocol involves the formation of caudal neural progenitors (days 1–5) and neurospheres (days 5–12). Neurospheres are plated down onto a monolayer (day 12) and then removed after 48 h (day 14), to enrich for NC cells. NC cells are transduced with the NGN2 and reverse tetracycline transactivator lentiviruses (day 14) and NGN2 expression is induced for 96 h (days 15–19) and successfully transduced cells are obtained *via* puromycin selection (days 17–19). Finally, proliferating cells are removed using an antimitotic agent (AraC; days 24–26) and the _NGN2_iSN are sampled for staining and RT-qPCR (day 34) or patch clamping (day 34–44). **(B–C)** Plating neurospheres for 48 h using the above protocol enriches for SOX10+ and P75^NTR^+ NC cells. Neurospheres were either disaggregated into single cells or plated as whole spheres in NM supplemented with 10 μM Y-27632 for 24 h or 48 h.** (B)** Representative immunocytochemistry images of the NC markers P75^NTR^ (green) and SOX10 (pink). **(C)** The percentage of SOX10 positive cells, *n* = 3 biological replicates, >300 cells counted per biological replicate, error bars presented as SEM, **p < 0.01, ***p < 0.001.

We modified the previously established protocol (Alshawaf et al., 2018) that uses a combination of small molecules and growth factors to generate a mixed population of sensory neurons from hPSCs (Figure 1A). Briefly, hPSC were first differentiated to caudal neural progenitor cells using small molecule inhibitors of the GSK3β (CHIR99021) and activin/nodal pathways (SB431542). Caudal neural progenitors were further differentiated to NC by treatment with BMP2 (Denham et al., 2015; Alshawaf et al., 2018; Abu-Bonsrah et al., 2019). To mimic the stages of sensory neurogenesis, this study set out to transiently induce NGN2 expression in migrating NC cells, defined by the expression of SOX10 and P75^NTR^. To enrich for the migrating NC population, neurospheres were plated onto a monolayer for 24 h or 48 h and compared to neurospheres that were disaggregated into single cells supplemented with Y-27632 (Figures 1B,C). Whole neurospheres plated for 24 h had a 5.9-fold increase in SOX10 expression (84 ± 6% SOX10+ cells) compared to disaggregated neurospheres (14 ± 1% SOX10+ cells; *t*_(4)_ = 11.47, *p* = 0.003, Figure 1C). Similarly, whole neurospheres plated for 48 h had a 3.5-fold increase SOX10 expression (66 ± 6% SOX10+ cells) compared to disaggregated neurosphere cultures (19 ± 3% SOX10+ cells; *t*_(4)_ = 7.54, *p* = 0.0017, Figure 1C). Furthermore, the 48 h cultures of whole sphere migrating cells also co-expressed the NC marker P75^NTR^. Additionally, PAX3/7 expression was detected in all conditions (Supplementary Figure 2). Thus, 48 h of migration was considered sufficient for the enrichment of SOX10+/P75^NTR^+ NC cells.

**Figure 2:**
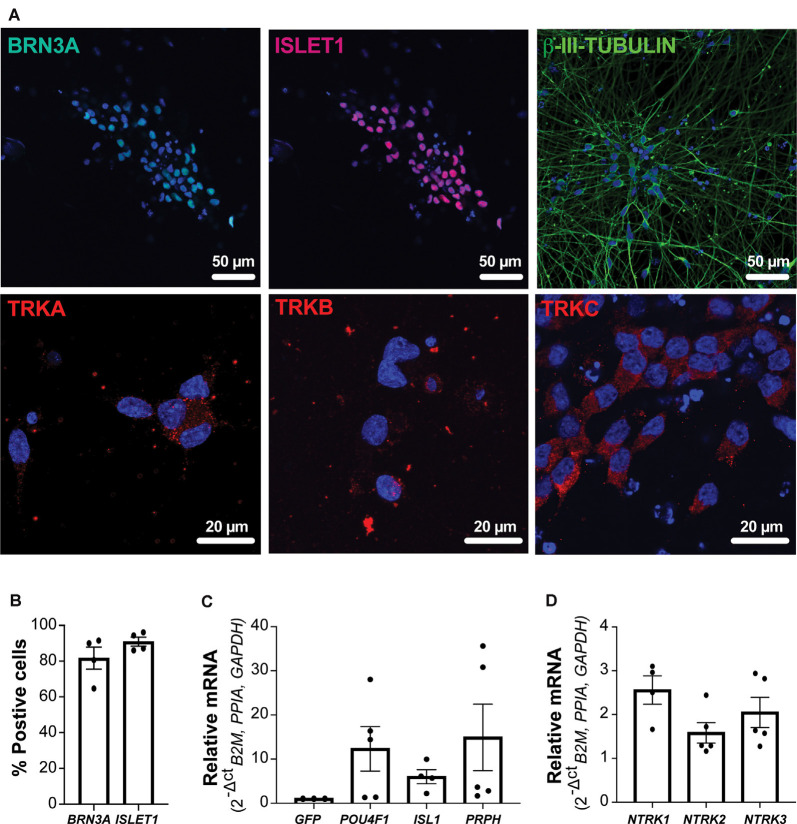
Protein and mRNA expression of sensory neuron markers in _NGN2_iSNs. **(A)**_NGN2_iSN was positive for the protein expression of the neuronal marker ß-III-TUBULIN (green) and the sensory neuron markers BRN3A (green), ISLET1 (red), TRKA (red), TRKB (red), and TRKC (red), nuclei shown in blue. **(B)** The percentage of neurons expressing either BRN3A or ISLET1, *n* = 4 biological replicates, >200 cells counted per biological replicate. **(C)** Fold change in mRNA expression of *POU4F1, ISL1*, and *PRPH* normalized the GFP control. **(D)** Relative mRNA expression of *NTRK1, NTRK2, NTRK3* (normalized to the house-keeping genes *B2M, PPIA*, and *GAPDH*), error bars presented as SEM, *n* = 5 biological replicates.

As outlined in Figure 1, NGN2 expression was induced in enriched migrating NC cells. Cultures were assessed by immunocytochemistry and RT-qPCR analyses at day 34 to determine the expression of sensory DRG neuronal markers. Expression of the neuronal marker ß-III-TUBULIN was evident throughout the cultures. Additionally, _NGN2_iSN expressed pan-sensory neuron markers, with 82% of neurons expressing BRN3A and 91% expressing ISLET1 (Figures 2A,B). Furthermore, BRN3A, ISLET1 (encoded for by *POU4F1* and *ISL1*, respectively), and *PRPH* mRNA transcript levels were increased in the _NGN2_iSN cultures relative to cultures transduced with a GFP control vector (Figure 2C). Additionally, neurons were positive for the nociceptor marker TRKA, the mechanoreceptor marker TRKB, and the proprioceptor marker TRKC (encoded for by *NTRK1, NTRK2, NTRK3*, respectively, Figures 2A,D). These data suggest that induced expression of NGN2 in NC progenitors resulted in their differentiation to heterogeneous populations of DRG sensory neurons and therefore are referred to as “NGN2-induced sensory neurons” (_NGN2_iSN).

### _NGN2_iSNs Excitability Profile

**Figure 3:**
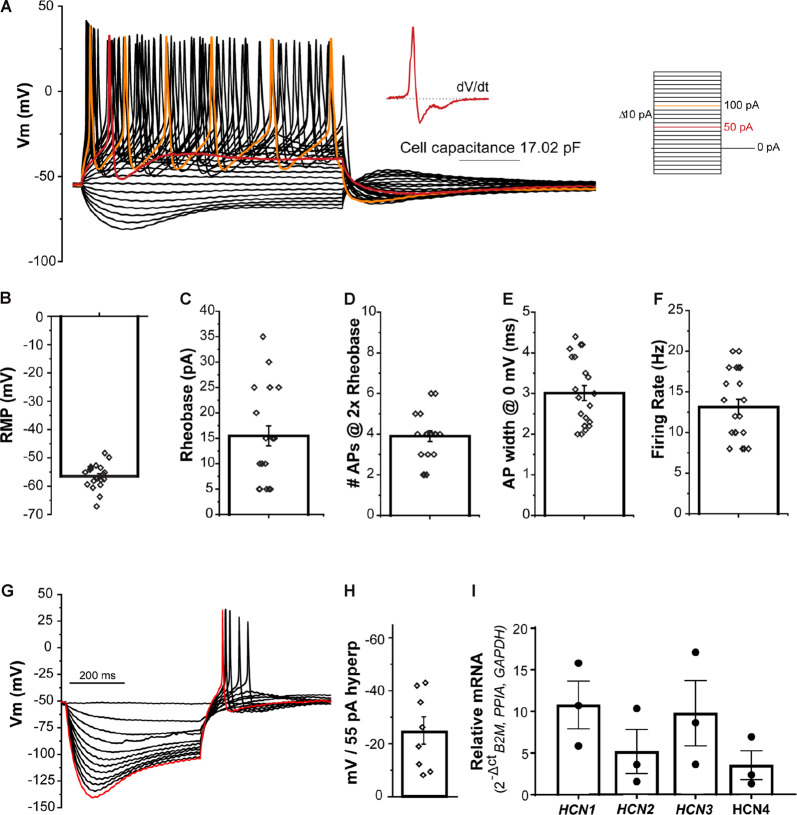
Excitability profile of _NGN2_iSN. **(A)** Current clamp recording of a 17 pF _NGN2_iSN. Representative membrane potential voltage responses recorded under current-clamp conditions elicited by progressive current injections (from −60 to +180 pA, Δ 10 pA, 500 ms). Traces corresponding to rheobase and 2× rheobase are highlighted in red and orange, respectively. Inset: first derivative of the trace at rheobase. **(B–F)** Summary of excitability properties: resting membrane potential (RPM, mV, **B**), rheobase (pA, **C**), number of fired action potential (APs at 2× rheobase, **D**), AP width (at 0 mV, ms, **E**), and AP firing rate (Hz, **F**). **(G)** Potassium-channel mediated membrane hyperpolarization upon current injections (0 to −55 pA, Δ 5 pA, 500 ms). The membrane potential traces in response to −55 pA current injection is highlighted in red.** (H)** Bar graph showing mV change in peak vs. steady-state current amplitude after a 55 pA hyperpolarizing pulse. **(I)** Relative mRNA expression of *HCN1-4* transcripts detected in _NGN2_iSN (normalized to the house-keeping genes *B2M, PPIA*, and *GAPDH*), error bars presented as SEM, *n* = 3 biological replicates.

The passive and active electrical properties of the _NGN2_iSN were characterized by current clamp electrophysiological recordings in the whole-cell patch-clamp recording configuration. Standard neuronal excitability parameters, including resting membrane potential (RMP, mV), rheobase (pA), number of APs at 2× rheobase (#APs @ 2× rheobase), AP width (AP width @ 0 mV, ms), and firing rate (Hz) were evaluated upon the establishment of the whole-cell configuration and in response to small current injections. Representative current-clamp recordings of _NGN2_iSNs are depicted in Figure 3A displaying robust tonic neuronal firing. These neurons had an average RMP of −56.4 ± 0.9 mV (*n* = 21, Figure 3B) and cell capacitance of 12.7 ± 1 pF (*n* = 21). Rheobase is defined as the current injection necessary to elicit a single AP and provides a measure of neuronal excitability. Firing of a single AP by the _NGN2_iSN shown in Figure 3A is highlighted in red (and presented as single trace “SN9” in Supplementary Figure 3 together with all other neurons included in the analysis). The average rheobase determined in these neurons was 15.5 ± 2.0 pA (*n* = 21, Figure 3C). Accordingly, injection of a current equivalent to twice rheobase (shown in orange in Figure 3A) elicited on average 3.9 ± 0.3 APs (*n* = 21, Figure 3D). The mean AP width measured at 0 mV was 3.0 ± 0.2 m (*n* = 21, Figure 3E). A maximal firing rate of 13.1 ± 1.0 Hz (*n* = 21, Figure 3F) was observed in this neuronal population. The first derivative (d*V*/d*t*) of the APs fired at rheobase for all cells included in this study (presented in Supplementary Figure 3) revealed that ~30% (6 out of 21) _NGN2_iSN displayed clearly defined humps arising from the inflection on the descending slope of the AP, which is typically associated with functional aspects of C-fiber nociceptive neurons in rodents (Ritter and Mendell, 1992).

The _NGN2_iSN displayed robust membrane hyperpolarization during current-clamp recordings as shown in the example presented (Figure 3G). Quantification of the hyperpolarization-activated current (*I_h_*), was obtained from measurement of the difference between the initial peak and the steady-state current during a 500 ms hyperpolarizing pulse of 55 pA revealing a potential change of −24.5 ± 5.1 mV (*n* = 8, Figure 3H). Hyperpolarization-activated cyclic nucleotide-gated (HCN) channels underlie *Ih* and are mediated by four major isoforms (HCN1-4). Accordingly, transcripts encoding all four family members were readily detected in our _NGN2_iSN cultures as evidenced by RT-qPCR (Figure 3I).

### Ionic Basis of _NGN2_iSN Excitability

The generation and conduction of APs are critical to sensory neuron function. Therefore, the ion channels underpinning neuronal excitability are key players in sensory biology. In rodents, distinct voltage-gated ion channel constellations support the unique intrinsic properties of the major DRG sensory neuron classes (Zheng et al., 2019). In this study, we used voltage clamp to perform a detailed investigation of the whole-cell voltage-dependent Na^+^, K^+^, and Ca^2+^ conductance components of the human _NGN2_iSN AP, as well as provide evidence of functional proton-activated currents (*I_pH_*) and its likely mediators.

#### Voltage-Gated Sodium Currents

Stem cell-derived neuronal cultures, such as those generated in this study, consist of an intricate network of cell bodies interconnected *via* long processes (Figure 1A, _NGN2_iSN). In these networks, poor space clamp hinders control of *I_Na_* from intact neurons, a challenge not experienced with primary DRG neurons, which are trimmed of long processes upon extraction and dissociation. Neuronal cell geometry affects the quality of electrophysiological recordings. The axon and neurites constitute different compartments from the neuron’s soma; hence, the voltage clamp of the cell body can fail to provide homogenous control of the membrane potential throughout the whole cell. Whole-cell currents from intact neurons under somatic voltage clamp contain a mixture of *I_Na_* from the cell body and axial current from escaped axonal spikes. We used the method described by Milescu et al. (2010) to inactivate axonal sodium channels as a means to isolate and adequately control somatic *I_Na_* from the axial current thus providing a more accurate characterization of *I_Na_*.

**Figure 4:**
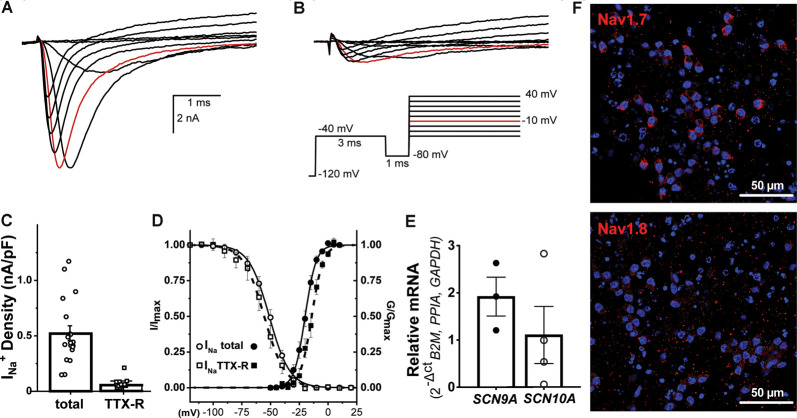
Voltage-gated sodium currents in _NGN2_iSN. Sodium currents (*I_Na_*) from _NGN2_iSN are predominantly mediated by TTX-S Nav channels. **(A,B)** Representative *I*_Na_ current traces in control **(A)** and the presence of 300 nM TTX **(B)** elicited by the voltage protocol shown in the inset. **(C)**_NGN2_iSN sodium current (*I*_Na_) density upon depolarization to −10 mV (highlighted in red in **A** and **B**) calculated from peak total and TTX-R *I_Na_* current in nA/pF. **(D)** Voltage- dependence of activation (filled symbols) and steady-state inactivation (SSI; empty symbols) of the total (●, ○) and TTX-R (300 nM TTX, ▪, □) components of *I_Na_*. **(E)** Relative mRNA expression of *SCN9A* and *SCN10A* transcripts in _NGN2_iSN (normalized to the house-keeping genes *B2M, PPIA*, and *GAPDH*), error bars presented as SEM, *n* = 3–4 biological replicates. **(F)** Immunoreactivity against human Nav1.7 and Nav1.8 antibodies.

Sensitivity to inhibition by the pufferfish alkaloid, tetrodotoxin (TTX), distinguishes two pharmacological families of voltage-dependent sodium channels (Navs). TTX sensitive (TTX-S) are those Nav isoforms blocked by low nM TTX whereas the TTX resistant (TTX-R) are represented by Nav isoforms inhibited by micromolar TTX exposure. The representative *I_Na_* families shown in Figure 4 were elicited by the pulse protocol described in the inset and were recorded in the absence (Figure 4A) and presence of 300 nM TTX (Figure 4B). We determined the contribution of each Nav-mediated component to the available total *I_Na_* from _NGN2_iSNs in the absence (control) and after exposure to 300 nM TTX. Ensemble current density estimated from peak *I_Na_* in response to a depolarizing pulse to −10 mV (red traces in Figures 4A,B) provides an estimate of the total *I_Na_* available in _NGN2_iSNs resulting in an average current density of 536.0 ± 54.0 pA/pF. The same measurements in the presence of 300 nM TTX revealed that ~13% of the total *I_Na_* remains, which is equivalent to a current density of 68.4 ± 10.5 pA/pF that is carried by TTX-R Nav channels in_ NGN2_iSNs (*n* = 19, Figure 4C). Peak current amplitude from *I_Na_* families in the absence (Figure 4A) and presence of 300 nM TTX (Figure 4B) were plotted as a function of the stimulus potential to determine the voltage-dependence of activation of the total (*I*_total_) and TTX-R (*I*_TTX-R_) components of _NGN2_iSNs *I_Na_* (Figure 4D). The total *I_Na_* activates with a half-activation potential (*V*_0.5_) of −19.9 ± 0.2 mV (●, *n* = 23, Figure 4D), whereas the TTX-R component does so with a *V*_0.5_ = −14.2 ± 0.3 (▪, *n* = 15, Figure 4D). SSI curves were generated by a 500 ms pre-pulse to potentials from −120 to +20 mV, in 10 mV increments (*V*_h_ −80 mV), followed by a test pulse to 0 mV (Figure 4D). Peak *I_Na_* amplitude measured upon the test pulse gauge the degree of Nav channel inactivation at the pre-pulse potentials. The bulk of the *I_Na_* inactivates with a *V*_0.5_ = −51.1 ± 0.3 mV (◯, *n* = 26, Figure 4D) whereas the TTX-R *I_Na_* in _NGN2_iSN displays an apparent SSI-*V*_0.5_ of −55.5 ± 0.4 mV (□, *n* = 16, Figure 4D).

At the molecular level, we explored *SCN9A* and *SCN10A* transcripts and their protein products Nav1.7 and Nav1.8 channels due to their apparent specific expression in sensory neurons. _NGN2_iSN had a high abundance of *SCN9A* and Nav1.7 expression, likely representing the TTX-S current and a lower proportion of *SCN10A* mRNA and Nav1.8 protein expression, consistent with a small contribution of the TTX-r current (Figures 4E,F).

#### Voltage-Gated Potassium Currents

Potassium currents (*I_K_*) are crucial regulators of neuronal excitability and homeostasis as they contribute to the RMP and membrane repolarization, thus they modulate the shape, duration, and frequency of APs. Voltage activated K^+^ (Kv) channels constitute the most diverse family with 40 Kv members organized in 12 subfamilies which can make physiological identification of ion channels in neurons challenging (Finol-Urdaneta et al., 2020). Yet, the properties and pharmacology of *I_K_* provide valuable information about sensory neuron functional subtypes (Giacobassi et al., 2020). To date, little information is available about the potassium channels expressed in stem cell-derived sensory neurons. A direct study by whole-cell patch-clamp recordings from _NGN2_iSNs was used to characterize its biophysical properties and their constituent ion channels.

**Figure 5:**
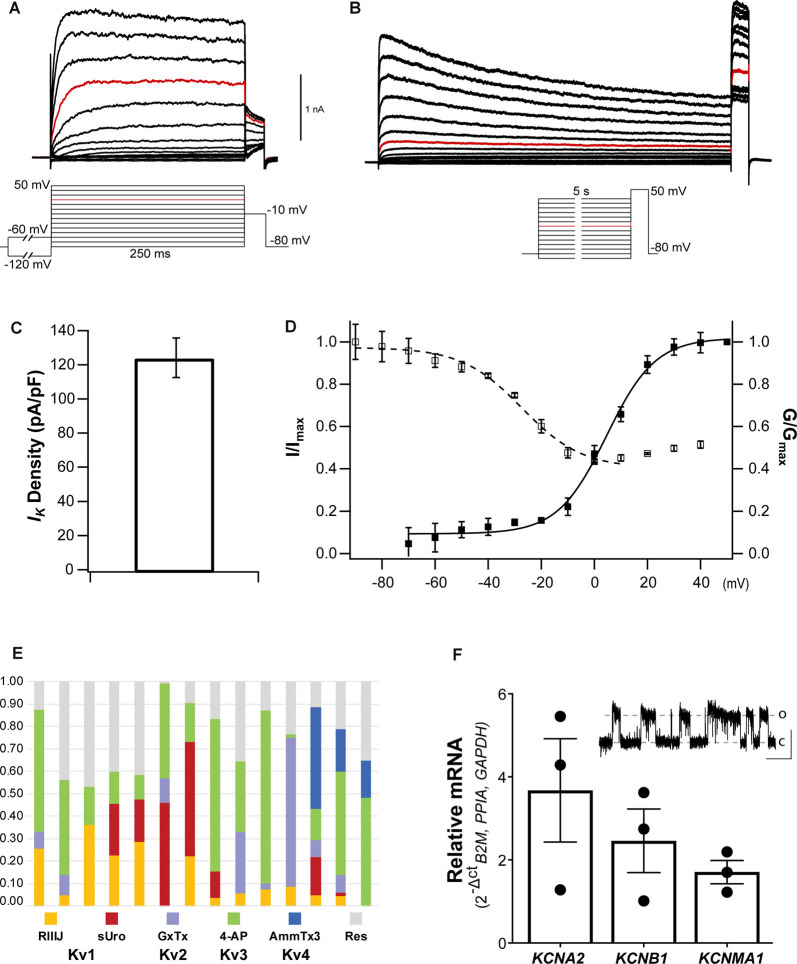
Voltage-gated potassium currents in _NGN2_iSN.** (A,B)** Representative delayed rectifier potassium currents (*I_K_*) recorded in a 14.56 pF cell upon standard activation (inset in **A**) and SSI (inset in **B**) protocols. **(C)** *I_K_* current density (pA/pF) quantification. **(D)** Voltage dependence of activation (▪) and SSI (□) of I_K_. **(E,F)** The molecular identity of _NGN2_iSN *I_K_*. **(E)** Stacked bar plots of 14 different _NGN2_iSNs showing the fraction of total outward *I_K_* current inhibited by inhibitors of Kv1 (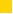 300 nM RIIIJ and 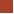 10 nM Urotoxin), Kv2 (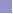 300 nM GxTx), Kv3 (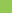 100 μM 4-AP), and Kv4 (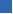 1 μM AmmTx3) channels. The fraction of *I_K_* remaining in the presence of all inhibitors is shown in gray (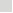 Res). **(F)** Relative mRNA expression of the transcripts encoding the major Kv channels mediating *I_K_* in sensory neurons (*KCNA2*, *KCNB1*, and *KCNMA1*). The inset displays cell-attached single-channel recordings of a large conductance potassium channel consistent with the functional expression of BK (~500 nM [Ca^2+^]_i_, pulse to +80 mV, scale bars: 4 pA, 500 ms, o, open; c, closed).

Upon establishing the whole-cell configuration, cells were held at a *V*_h_ of −80 mV. Typical whole-cell depolarization-activated *I_K_* recordings are shown in Figures 5A,B. The whole-cell *I_K_* from _NGN2_iSN somatic recordings were large in virtually all cells recorded with patch electrodes of 2–4 MΩ resistance, yielding significant outward currents with peak amplitudes of ~1.5 nA (at +20 mV, highlighted in red in Figure 5A). *I_K_* density was estimated from peak *I_K_* recorded at +20 mV normalized to the cell capacitance resulting in an average *I_K_* density of 124.5 ± 11.7 pA/pF (*n* = 29, Figure 5C). A pre-pulse step to −60 mV or −120 mV (inset in Figure 5A) was implemented to differentiate between slow inactivating (delayed rectifier) and fast inactivating (A-type) *I_K_* components, respectively. However, within the same _NGN2_iSN, currents elicited by either protocol had indistinguishably slow kinetics suggesting a low contribution from fast inactivating Kv channels to the total *I_K_* in these cells. Thus, under our experimental conditions, the overwhelming majority of the elicited *I_K_* (Figure 5A) displayed characteristics consistent with those mediated by delayed rectifier Kv channels.

The voltage dependence and kinetics of activation and inactivation of _NGN2_iSN somatic *I_K_* were determined using the protocols described in the insets below the example families of outward *I_K_* traces (insets under Figures 5A,B for activation and inactivation, respectively). Instantaneous current-voltage (I-V) curves were well-fitted with a single, first-order Boltzmann function, yielding a mean *V*_0.5_ of 4.6 ± 1.1 mV and slope factor (*ka*) of 9.2 ± 0.9 (▪, *n* = 16, Figure 5D). Kv mediated currents were observed at potentials above −60 mV (Figure 5D), however, the threshold for voltage-activated *I_K_* was around −30 mV. _NGN2_iSN rapid and voltage-dependent *I_K_* activation kinetics are well described by a single exponential function. Thus, the mean activation time constant for a voltage step from *V*_h_ to +20 mV was 8.1 ± 2.4 ms (*n* = 10) and decreased exponentially with voltage to 4.6 ± 0.9 ms (*n* = 10) for the step to +60 mV. _NGN2_iSN *I_K_* inactivation was slow and only appreciable during long stimulation protocol. The voltage dependence of inactivation was examined using the voltage protocol shown in the inset with marked current decay becoming evident during the 5 s long voltage steps (Figure 5B). A single Boltzmann function was used to describe the voltage dependence of inactivation of the major component as other contributors were too small to be accurately defined under our experimental conditions (Figure 5D). The midpoint of the voltage dependence of inactivation was −27.4 ± 2.2 mV and slope factor (*k*) of 11.3 ± 2.02 (□, *n* = 14, Figure 5D).

Pharmacological assessment with selective inhibitors and venom-derived peptides constitutes a useful “dissection of the current” approach to identify individual mammalian voltage-gated potassium channels. The contribution of various Kv channels to *I_K_* in _NGN2_iSNs is largely dependent on their kinetics and voltage-dependent activation and inactivation and therefore may have a maximal relative contribution to the whole-cell Kv currents at different time points and voltages. Fourteen individual _NGN2_iSN were surveyed for potential candidates underpinning *I_K_* by quantifying the fraction of current inhibited by a sequence of Kv channel inhibitors cumulatively applied at concentrations and order devised to enhance selective inhibition (Figure 5E). We began by applying 300 nM κ-conotoxin RIIIJ as this peptide is ~10–100-fold more potent against heteromeric than homomeric Kv1 channels and observed that 12 of 14 cells, displayed a RIIIJ-sensitive component (3–35%) consistent with the expression of heteromeric channels containing Kv1.2 and likely Kv1.1 or Kv1.6 subunits (Cordeiro et al., 2019). The scorpion peptide Urotoxin (10 nM) further blocked 2–46% of the outward *I_K_* evidencing functional expression of Kv1.2 homomeric channels in 50% of the tested cells (7/14). The selective Kv2 channel spider toxin Guanxitoxin (300 nM) was used to evidence participation of Kv2 channels and observed a GxTx-sensitive component in 8/14 _NGN2_iSNs. A 4-aminopyridine (4-AP; 100 μM) sensitive component, ranging from 11% to 77% of the total *I_K_*, was present in all cells tested highlighting the contribution of Kv3 channels that was evidenced by sequential application of this small molecule. As a final step, 1 μM AmmTx3 identified the presence of Kv4 channels, likely associated with the accessory dipeptidyl peptidase-like proteins (DPP) 6 and 10, in 3/14 cells. Interestingly, the cumulative application of all these Kv channel inhibitors was not sufficient to eliminate all the voltage-dependent outward *I_K_* in most (13/14) _NGN2_iSNs suggesting a substantial (9–47%), yet unidentified, component of *I_K_* in human stem cell-derived sensory neurons.

RT-qPCR (or pharmacological) analysis of all potential potassium channel isoforms in our cultures was beyond the scope of this work. A scan for representative transcripts encoding for Kv channels commonly expressed in mammalian DRG sensory neurons revealed the presence of *KCNA2, KCNB1*, and *KCNMA1* mRNA encoding for Kv1.2, Kv2.1, and BK channels, respectively in total _NGN2_iSN RNA isolates (Figure 5F). The higher relative expression of *KCNA2* transcripts is in agreement with the identification of RIIIJ- and Urotoxin-sensitive components; whereas the relative abundance of *KCNB1* mRNA is consistent with the observed GxTx-sensitive component in _NGN2_iSNs *I_K_*.

In the whole-cell recording configuration, the presence of EGTA (5 mM) in the patch pipette allows little to no contribution from Ca^2+^-activated potassium currents to the total *I_K_*. Nevertheless, we observed the appearance of a 295.9 ± 96.4 pS (at +80 mV) potassium channel conductance with an open probability of 0.42 [intracellular (Ca^2+^) ~500 nM] in cell-attached somatic patches of _NGN2_iSN. These single-channel recordings, observed in six out of 23 cells, are suggestive of the functional expression of BK channels and consistent with the detection of *KCNMA1* transcripts in our neuronal cultures.

#### Voltage-Gated Calcium Currents

**Figure 6:**
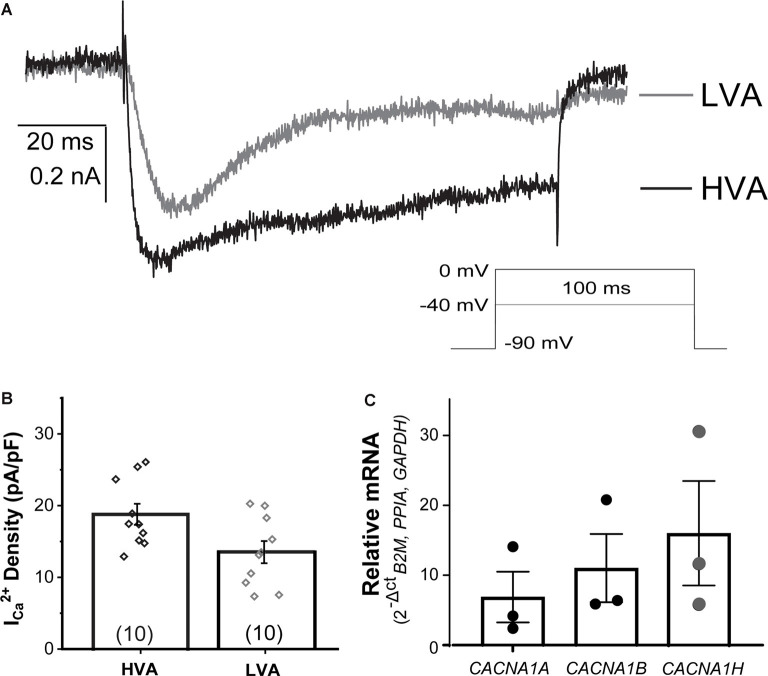
Voltage-gated calcium currents (*I_Ca_*) present in _NGN2_iSN. _NGN2_iSN *I_Ca_* evidence contribution from high and low voltage-activated calcium channels (HVA and LVA, respectively). **(A)** Representative HVA at 0 mV (black) and LVA at −40 mV (gray) *I_Ca_* traces. Stimulation protocol is shown in the inset (100 ms Vh −90 mV, 0.2 Hz). **(B)** Bar graph summarizing *I_Ca_* current density (pA/pF) of the HVA and LVA mediated components. **(C)** Relative mRNA expression of transcripts encoding the major calcium channels mediating HVA (*CACNA1A*, *CACNA1B*) and LVA (*CACNA1H*) in _NGN2_iSN (normalized to the house-keeping genes *B2M, PPIA*, and *GAPDH*), error bars presented as SEM, *n* = 3 biological replicates.

In dissociated rodent and human DRG neurons, *I_Ca_* is mediated by different voltage-dependent Ca^2+^ channels (L-, N-, P/Q-, R-type, and T-type channels), whose expression may be related to specific somatosensory cell types. In _NGN2_iSNs, low (LVA or T-type) and high voltage-activated (HVA, all other isoforms) *I_Ca_* (Figure 6) could be readily identified in whole-cell recordings using external solutions designed to isolate Ca^2+^-mediated ionic currents. Representative LVA (gray) and HVA (black) *I_Ca_* were elicited from a holding potential of −90 mV to a test pulse to −40 or 0 mV, to isolate LVA or HVA *I_Ca_*, respectively, for 100 ms at a frequency of 0.1 Hz (Figure 6A). The average current densities measured for LVA were slightly smaller than HVA (LVA *I_Ca_* = 13.4 ± 1.5 pA/pF, *n* = 10; HVA *I_Ca_* = 18.8 ± 1.5 pA/pF, *n* = 10, Figure 6B). The predominant channels carrying LVA *I_Ca_* (Cav3.2/CACNA1H), and HVA *I_Ca_* (Cav2.1/*CACNA1A* and Cav2.2/*CACNA1B*) in rodent DRG neurons were detected in human _NGN2_iSN by RT-PCR analyses (Figure 6C).

#### Proton-Activated Currents

Proton-activated currents have been historically associated with sensory neurons, its mediators the acid-sensing (ASIC1-4) and transient receptor potential vanilloid (TRPV) channels are well-established modulators of mechanosensation, nociception, and proprioception (Davis et al., 2000; Walker et al., 2003; Gu and Lee, 2010; Omerbašić et al., 2015). Therefore, we explored the activation of these ionic currents in the _NGN2_iSN generated in this study by a drop in extracellular pH and their sensitivity to amiloride to distinguish between ASIC and TRPV1 mediated proton-sensitive currents.

**Figure 7:**
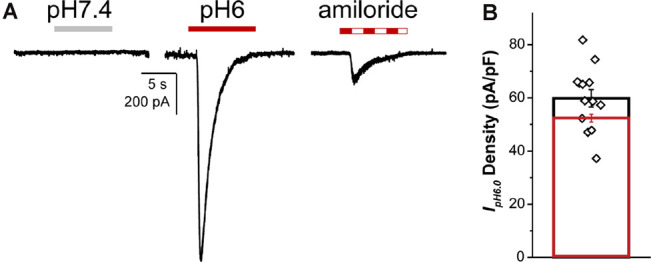
_NGN2_iSN proton- activated currents. _NGN2_iSN *I_pH6.0_* evidences contribution from ASIC channels. **(A)** Current trace at pH 7.4 (gray bar), representative inward currents upon rapid change to pH 6.0 drop (red bar) and pH 6.0 drop in the presence of 10 μM amiloride (dashed bar; *V*_h_ = −80 mV). **(B)** Bar graph summarizing *I_pH6.0_* current density (pA/pF) of the total (black, *n* = 13) and the 10 μM amiloride-sensitive current (red, *n* = 6).

Whole-cell proton-activated currents were elicited by rapidly decreasing the extracellular solution pH from 7.4 to 6.0 (*I_pH6.0_*). Patch-clamp recordings of _NGN2_iSNs proton currents in control and in the presence of the heterotrimeric sodium (ENaC) channel blocker, amiloride, are shown in Figure 7A. The drop of extracellular pH-induced transient inward currents whose maximal amplitude was used to determine the overall _NGN2_iSN *I_pH6.0_* density resulting in 59.8 ± 3.31 pA/pF (*n* = 13, Figure 7B). In sensory neurons, proton activated current can be mediated by TRPV1 or any of the ASIC family channels. In the presence of 10 μM amiloride, ~15% of *I_pH6_* remained (% block 86.9 ± 1.19%, *n* = 6, Figure 7) in the _NGN2_iSNs tested consistent with relatively low abundance of TRPV1 protein and transcripts detected in our cultures by immunofluorescence and qPCR (Supplementary Figure 4). At the concentration used, amiloride fully inhibits ASIC channels whilst sparing TRPV1 thus suggesting a sizeable contribution of ASIC-mediated currents to the proton sensitive conductance in human _NGN2_iSN cells.

## Discussion

This study describes the generation and functional characterization of human sensory neurons derived from hPSCs. We optimized a protocol that results in abundant electrically excitable cells capable of firing regenerative APs. The biophysical and pharmacological properties of voltage-dependent ionic currents in _NGN2_iSNs were investigated and related to those known to be present in mammalian primary sensory neurons. The present study represents the first detailed biophysical and pharmacological characterization of the different conductances supporting excitability in human sensory neurons and gives insights into the molecular identities of native ionic currents.

We have optimized the generation of functionally mature sensory neurons from hPSCs by enriching our cultures for NC cells and then inducing the expression of the transcription factor NGN2. NC enrichment was enhanced by plating whole neurospheres in the presence of the ROCK inhibitor Y-27632 instead of disaggregating the spheres into single cells. Plating whole spheres results in a significantly higher proportion of SOX10+ cells, a key marker for migratory cells, compared to the dissociation into single cells approach (66% vs. 19%, respectively). The increased proportion of SOX10+ cells is attributed to the inhibition of the ROCK pathway, which was shown to promote the delamination and differentiation of NC cells from the developing neural tube (Groysman et al., 2008) and effectively increases the proportion of NC cells during hPSC differentiation (Hotta et al., 2009; Kim et al., 2015). Interestingly, SOX10+ cells appeared after just 24 h of neurosphere plating, whereas P75^NTR^ positive cells were first observed after 48 h of plating. This is consistent with SOX10 expression spanning various stages of NC development (e.g., from the progression of NC precursors to migrating crest cells), with P75^NTR^ predominantly co-expressed with SOX10 in the migratory cells (McKeown et al., 2005; Betters et al., 2010; Curchoe et al., 2010). The expression of PAX3/7, which are transcription factors commonly expressed throughout NC development and specifically in pre-migratory and migratory NC cells (Basch et al., 2006; Betters et al., 2010; Maczkowiak et al., 2010; Murdoch et al., 2012), was also noted in the cultures at varying levels. Thus, we surmise that our 48 h cultures, which contain SOX10/P75^NTR^ positive cells, most likely represent migrating NC cells enriched culture in which the expression of NGN2 further drives the differentiation to functionally mature sensory neurons. Immunoreactivity towards key protein markers confirms the successful differentiation of sensory neurons following NGN2 expression verified by immunofluorescence confocal microscopy. A significant proportion of _NGN2_iSNs derived from the described protocol was positive for the pan-sensory neuron markers BRN3A and ISLET1 (~80–90% positive), which is notably higher than previous protocols not implementing NGN2 expression in NC cells (~5% BRN3A^+^/ISLET1^+^ cells; Alshawaf et al., 2018). The high proportion of BRN3A expression throughout our cultures is qualitatively similar to previously described differentiation protocols inducing the expression of different combinations of transcription factors, such as BRN3A, NGN1, and NGN2 to generate sensory neurons. However, in contrast to previous reports (Blanchard et al., 2015, Schrenk-Siemens et al., 2015) we quantified BRN3A expression within the whole culture (e.g., without excluding non-neuronal cells). To address cellular heterogeneity within the culture dish and avoid potential bias, the number of BRN3A^+^ neurons was normalized to the total cell count within the dish. Consequently, the differentiation strategy presented in this work generated 82% BRN3A^+^ cells, thus evidencing an enrichment for sensory neurons and a significantly low representation of non-neuronal phenotypes. Detection at the protein and transcript level, of the three major sensory neuron markers TRKA, TRKB, and TRKC in our sensory neuron cultures suggest that our differentiation protocol generates a heterogeneous population of sensory neurons, including nociceptors, mechanoreceptors, and proprioceptors. In general, *in vitro* stem cell differentiation cannot fully replicate all of the differentiation cues found *in vivo*, particularly in terms of establishing the proportion of cell types within a tissue or ganglia, such as the DRG. This is partly due to the challenge of replicating *in vitro* the timing of NC migration and differentiation that occurs *in vivo*, which gives rise to the different sensory neuronal populations. The heterogeneity of sensory subtypes found within the _NGN2_iSN cultures underscores the need for further work to delineate strategies aimed at enriching specific sensory subtypes and in-depth profiling of each of those subtypes. In this study, we used the transcription factor-mediated approach to direct crest differentiation to DRG sensory neurons. This approach can certainly be further developed to direct sensory neuronal differentiation to a specific phenotype/lineage if the specifying transcription factors for that lineage are known. Importantly, since the _NGN2_iSN cultures consist of heterogeneous sensory neuronal populations, these cultures represent an excellent model of human DRG neurons that can be used to further study the human sensory system and develop therapies for related sensory pathological conditions.

The use of molecular analyses, such as immunocytochemistry and RT-qPCR, are important in determining the success of differentiating hPSC to sensory neurons. However, the functional excitability profile of the generated neurons must be investigated to provide a reliable functional baseline for the model’s applicability in the investigation of diseases or the effect of drugs on defined ion channels. Functional profiling of hPSC-derived sensory neurons has primarily been focused on protocols that generate nociceptors (Chambers et al., 2012; Young et al., 2014; Blanchard et al., 2015; Eberhardt et al., 2015; Wainger et al., 2015; McDermott et al., 2019; Meents et al., 2019; Schoepf et al., 2020), rather than a heterogeneous population of sensory neurons akin to a more physiologically relevant model in which diverse populations of sensory neurons coexist. In this study, we sought to profile the spontaneously resulting mixed population of DRG sensory neurons at the transcriptional and functional levels including a detailed description of _NGN2_iSN excitability patterns and their underlying voltage-dependent conductances.

Our _NGN2_iSNs display phasic firing at relatively low current injections (average rheobase: 15 pA) that become tonic adapting AP firing of increasing frequency (max firing rate 13 Hz) with subsequently stronger stimulation, but no spontaneous firing under our experimental conditions. The observed heterogeneous electrophysiological signatures include repetitive low-frequency firing in response to current injection, delayed firing with long latency to the first AP followed by repetitive spiking, various patterns of firing adaptation as well as cells with “humped” AP (Supplementary Figure 3). As the major determinant of neuronal excitability, the RMP of the _NGN2_iSN generated with the described protocol had RMPs (Figure 3B, −56.4 ± 0.9 mV, *n* = 21) comparable to those found in dissociated human sensory neurons from young adults (mean age 18.2 years old) in culture (−62.4 ± 2.0 mV, *n* = 133; Two-tailed *P*-value = 0.2375; Davidson et al., 2014). These features highlight the suitability of our _NGN2_iSN cultures for modeling human sensory neuron function, its pharmacological modulation, and pathophysiology at the population level rather than a specific subtype.

The excitability of the different modalities of sensory neurons is shaped by unique patterns of ion channel expression. HCN channels mediate the excitatory hyperpolarization-activated current often associated with the repetitive neuronal electrical activity (Sartiani et al., 2017). Current clamp recordings from _NGN2_iSNs displayed hallmark features of *Ih* such as a voltage sag during hyperpolarizing current injection followed by rebound excitation upon current termination (Mayer and Westbrook, 1983; Robinson and Siegelbaum, 2003; Poolos, 2004). Consistent with studies of *Ih* in rodent DRG neurons (Kouranova et al., 2008), voltage sag onset was slow, appeared at potentials negative to −55 mV, and often led to AP firing upon rebound. The observed slow *Ih* onset and relative expression of *HCN3* transcripts detected in the _NGN2_iSN cultures are consistent with the functional properties of slow-gating HCN3 channels recorded in recombinant systems (Stieber et al., 2005). Moreover, we observed *HCN1* transcripts in our _NGN2_iSN cultures in agreement with recently published multiplex RNAscope *in situ* hybridization data from human DRG neurons of all sizes (Shiers et al., 2020). An in-depth biophysical characterization aided by pharmacological tools of human primary sensory neurons will warrant a better understanding of the homo- and heteromeric HCN channels complement present in these cells.

The development of neuronal excitability involves the coordinated expression of different voltage-gated ion channels. To define the molecular basis of _NGN2_iSN excitability, we dissected the major voltage-dependent conductances underlying the AP in human sensory neurons. We focused on the two major classes of voltage-dependent *I_Na_* studied in sensory neurons, which are broadly represented by the low threshold, rapidly activating and inactivating TTX-S Nav channels, and by the high threshold, more slowly activating and inactivating TTX-R Nav isoforms. We observed a healthy complement of *I_Na_* in _NGN2_iSN mediated largely (~90%) by TTX-S channels. Accordingly, the biophysical properties of the total *I_Na_* (activation *V*_0.5_ −19.9 mV, SSI *V*_0.5_ −51 mV) closely approximate those of human DRG TTX-S *I_Na_* (TTXs activation *V*_0.5_ −15 mV, SSI −58 mV, Zhang et al., 2017). The voltage dependence of activation of _NGN2_iSN TTX-R *I_Na_* is similar to that of dissociated human primary DRG neurons (_NGN2_iSN TTX-R *I_Na_* activation *V*_0.5_ −14.2 mV vs. human DRG TTX-R activation *V*_0.5_ −10 mV, Zhang et al., 2017). However, the SSI of the TTX-R *I_Na_* recorded in _NGN2_iSN is significantly left-shifted (SSI *V*_0.5_ −55 mV) in comparison to human DRG reported by Zhang et al. (2017; SSI *V*_0.5_ −20 mV).

The TTX-S Nav1.7 channel is typically associated with all sensory neuron subtypes and *SCN9A* mRNA is reported in virtually all neurons of human DRG (Ho and O’Leary, 2011; Haberberger et al., 2019; Shiers et al., 2020). We detected *SCN9A* transcripts in total RNA isolates and immunoreactivity with the Nav1.7 antibody, whilst observed low immunoreactivity towards Nav1.8, which is typically found in nociceptors. These, together with comparatively low TRKA staining in our cultures suggests that our protocol does not generate a high proportion of nociceptors and that other TTX-S Nav channels such as Nav1.6 and Nav1.1, which are abundantly detected in rodent mechano- and proprio-sensory neurons (Zheng et al., 2019), are also likely mediating the _NGN2_iSN TTX-S *I_Na_*. A small but readily observable TTX-R *I_Na_* component mediated by Nav1.8 was identified in these cultures. In human DRG, Nav1.8 immunoreactivity has been reported in several classes of sensory neurons, such as nociceptors and rapidly adapting Aβ low threshold mechanoreceptors (Aβ-LTM) surrounding hair follicles in human skin (Coward et al., 2000; Shields et al., 2012). Here, we used cell morphology and apparent membrane fitness to guide our patch-clamp characterization, resulting in a potential bias towards comparatively larger neurons in the cultures which could imply a higher representation of mechanosensory neurons in our recordings.

Voltage-dependent potassium currents have a pivotal role in the regulation of neuronal firing properties, such as the RMP, AP firing threshold, as well as AP shape and frequency. Importantly, differential modulation of *I_K_* can determine the response modality of their hosting neurons (González et al., 2017). The sheer abundance and diversity of K^+^ channels are a reflection of their relevance but also a daunting obstacle in their study at the physiological level (Cordeiro et al., 2019; Finol-Urdaneta et al., 2020; Giacobassi et al., 2020). A significant body of work on the various voltage-gated *I_K_* present in rodent DRGs exists, yet little, if anything, is known regarding the various K^+^ conductances present in human sensory neurons. This work on human _NGN2_iSN neurons provides a starting point to explore this important family of channels. Based on current kinetics, neuronal *I_K_* is mediated by fast inactivating (*I_KA_*) and slow inactivating delayed rectifying (*I_KDR_*) Kv channels. Hyperpolarizing pre-pulse protocols failed to demonstrate the presence of fast inactivating currents (not shown), therefore, we conclude that most of the _NGN2_iSN neurons analyzed here express large delayed rectifying outward currents. Several components of *I_K_* can be differentiated in patch-clamp recordings. Given the similarities in current kinetics and their activation/inactivation thresholds, we performed a pharmacological survey to approximate major molecular families mediating Kv currents and detected selected channel transcripts by RT-qPCR. All neurons recorded had a 4-AP sensitive component, consistent with the expression of slow inactivating members of the Kv3 family, whose principal physiological role involves the regulation of neurotransmitter release (Kaczmarek and Zhang, 2017). Interestingly, the transient presence of Kv3 family channels at the earliest stages of the rat and chicken nervous system development suggests that these channels may have a developmental role apart from those regulating firing rates and neurotransmitter release in adult neurons (Kaczmarek and Zhang, 2017). Future work warrants investigation of the relevance of Kv3 channel isoforms expression in identified subpopulations of human DRG neurons.

Sensitivity to conotoxin κ-RIIIJ (300 nM) in ~85% of cells tested revealed the functional expression of heteromeric Kv1.2-Kv1.1/Kv1.6 channels suggesting parallels with mouse L1 proprioceptive neurons (Giacobassi et al., 2020). Block by the Kv1.2-selective scorpion peptide Urotoxin was used to isolate the presence of homomeric Kv1.2 channels (Luna-Ramirez et al., 2020), which could be seen in half of the neurons screened consistent with abundant detection of *KCNA2* mRNA in our cultures. Clusters of Kv1.2 channels have been identified at the juxtaparanodes in co-cultures of human-induced pluripotent stem cell-derived sensory neurons and rat Schwann cells (Clark et al., 2017) as well as in rodent L2 Aδ-LTMR neurons (Giacobassi et al., 2020).

Detection of *KCNB1* transcripts by RT-qPCR and inhibition by spider peptide Guanxitoxin (Liu and Bean, 2014) are indicative of the expression of Kv2 channel family members in 57% of cells. In humans, *KCNB1* transcripts are present in 85.4% of small and large diameter DRG sensory neurons (Shiers et al., 2020), whereas Kv2 channels are expressed in all modalities of rodent mechanoreceptors (Zheng et al., 2019). In contrast to the widespread expression of the Kv4 family in rodent DRG (Na-Phuket and Covarrubias, 2009; Zheng et al., 2019), we observed Kv4 channel inhibition by AmmTx3 (Pathak et al., 2016) only in a small subset of the _NGN2_iSNs analyzed. Taken together, we found considerable variation in Kv currents within our cultures yet *I_KDR_* was the dominant outward current component of human _ NGN2_iSN *I_K_*.

Each DRG neuronal subtype exhibits a unique complement of voltage-gated ion channels that shapes the transduction of sensory information. Voltage-gated Ca^2+^ (Cav) channels activation enables the exocytosis of neurotransmitter-filled synaptic vesicles hence they are critically important to neuronal sensory function (Park and Luo, 2010). Cav channel currents mediated by the LVA and HVA Cav channel families were recorded electrophysiologically, as well as their encoding mRNAs _NGN2_iSNs. Transcripts encoding the α-subunits of (HVA) P/Q isoform Cav2.1 and N-type Cav2.2 HVA channels were identified, consistent with transcriptomic analyses of mouse sensory neurons (Zheng et al., 2019). Cav channel function in the human DRG remains unexplored, however, consistent with our RT-qPCR data, expression of *CACNA1B* mRNA (Cav2.2) has been reported in dissociated human DRGs (Castro et al., 2017). _NGN2_iSN display robust LVA *I_Ca_* akin to those mediated by the T-type calcium channel family. The magnitude of LVA currents in _NGN2_iSNs and the relatively low abundance of TRPV1 signal detected (immunofluorescence and qPCR) here suggest a parallel between our cultures and previously described subpopulations of capsaicin insensitive DRG and trigeminal neurons from adult mice (Pearce and Duchen, 1994; Borgland et al., 2001; Fioretti et al., 2011). It is of note, however, that capsaicin inhibits LVA calcium channels indirectly *via* calcium influx through TRPV1 (Comunanza et al., 2011) in DRG neurons and in HEK293 cells co-expressing Cav3.1 and TRPV1 (McArthur et al., 2019). Furthermore, capsaicin also directly inhibits all T-type channel isoforms with IC_50_s ~20 μM (McArthur et al., 2019) thus suggesting that the magnitude of T-type calcium currents may be underestimated in the presence of capsaicin.

RT-qPCR analysis of _NGN2_iSN evidence expression of transcripts encoding for TRKB receptor (encoded by *NTRK2*) and the T-type Cav3.2 (encoded by *CACNA1H*). The latter is regarded as a selective marker of Aδ- and C-low-threshold mechanoreceptors (LTMRs) innervating mouse skin hair follicles where Cav3.2 activation regulates light-touch and pain perception (Rutlin et al., 2014; François et al., 2015) supporting the generation of low threshold mechanoreceptors, as well as other sensory neurons using the hereby described protocol.

ASICs have a primary role in monitoring changes in extracellular acidosis caused by inflammation and injury and are also involved in the detection and transduction of mechanical stimuli (Price et al., 2000; Page et al., 2005; Deval et al., 2008; Gu and Lee, 2010; Geffeney and Goodman, 2012; Lin et al., 2016; Cheng et al., 2018). ASIC expression has been detected in nociceptors, mechanoreceptors, and proprioceptors, albeit with different subunit expression patterns depending on the sensory neuron subtype (Flegel et al., 2015; Usoskin et al., 2015; Lin et al., 2016; Papalampropoulou-Tsiridou et al., 2020). Thus, we investigated the presence of proton-sensitive currents and the expression of ASICs in our _NGN2_iSNs. A rapid decrease in pH elicited large inward currents, which was then inhibited by low concentrations of the pan-ASIC blocker, amiloride, in our _NGN2_iSNs. This functional data indicate the presence of functional ASICs in _NGN2_iSNs. Interestingly, ~10% of the proton-activated current was insensitive to amiloride suggesting the contribution from other proton-gated channels.

## Conclusion

This study describes the differentiation of hPSCs into functional sensory neurons, *via* a combined small molecule and transcription factor expression approach. The _NGN2_iSNs expressed sensory neuron markers and exhibited robust AP firing supported by voltage-gated Na^+^, K^+^, and Ca^2+^ conductances. Our characterization of stem cell-derived sensory neurons sheds light on the molecular basis of human sensory physiology and highlights the suitability of using hPSC-derived sensory neurons for modeling human DRG development and their potential in the study of human peripheral neuropathies and drug therapies.

## Data Availability Statement

The raw data supporting the conclusions of this article will be made available by the authors, without undue reservation.

## Ethics Statement

The studies involving human participants were reviewed and approved by University of Wollongong Human Ethics Committee. Written informed consent for participation was not required for this study in accordance with the national legislation and the institutional requirements.

## Author Contributions

AJH, JRM, RKF-U, and MD conceived and designed the study. AJH performed the cell culture and molecular profiling of the _NGN2_iSN, analyzed expression data and presentation. JRM and RKF-U performed functional characterization, analysis, and presentation. SMa performed molecular profiling of the _NGN2_iSN. SMi provided technical assistance. MD, LO, and DJA provided resources, reagents, supervision, and edited drafts of the manuscript. AJH, JRM, and RKF-U prepared the manuscript with comments and edits from all authors. All authors contributed to the article and approved the submitted version.

## Conflict of Interest

The authors declare that the research was conducted in the absence of any commercial or financial relationships that could be construed as a potential conflict of interest.
