# Supplementary material for: Molecular and Functional Characterization of Neurogenin-2 Induced Human Sensory Neurons
Source: Front Cell Neurosci. 2020 Dec 4;14:600895. doi: 10.3389/fncel.2020.600895 (PMC7761588; doi:10.3389/fncel.2020.600895)
Supplement: Supplementary file 1 [file Data_Sheet_1.pdf]

Supplementary Material

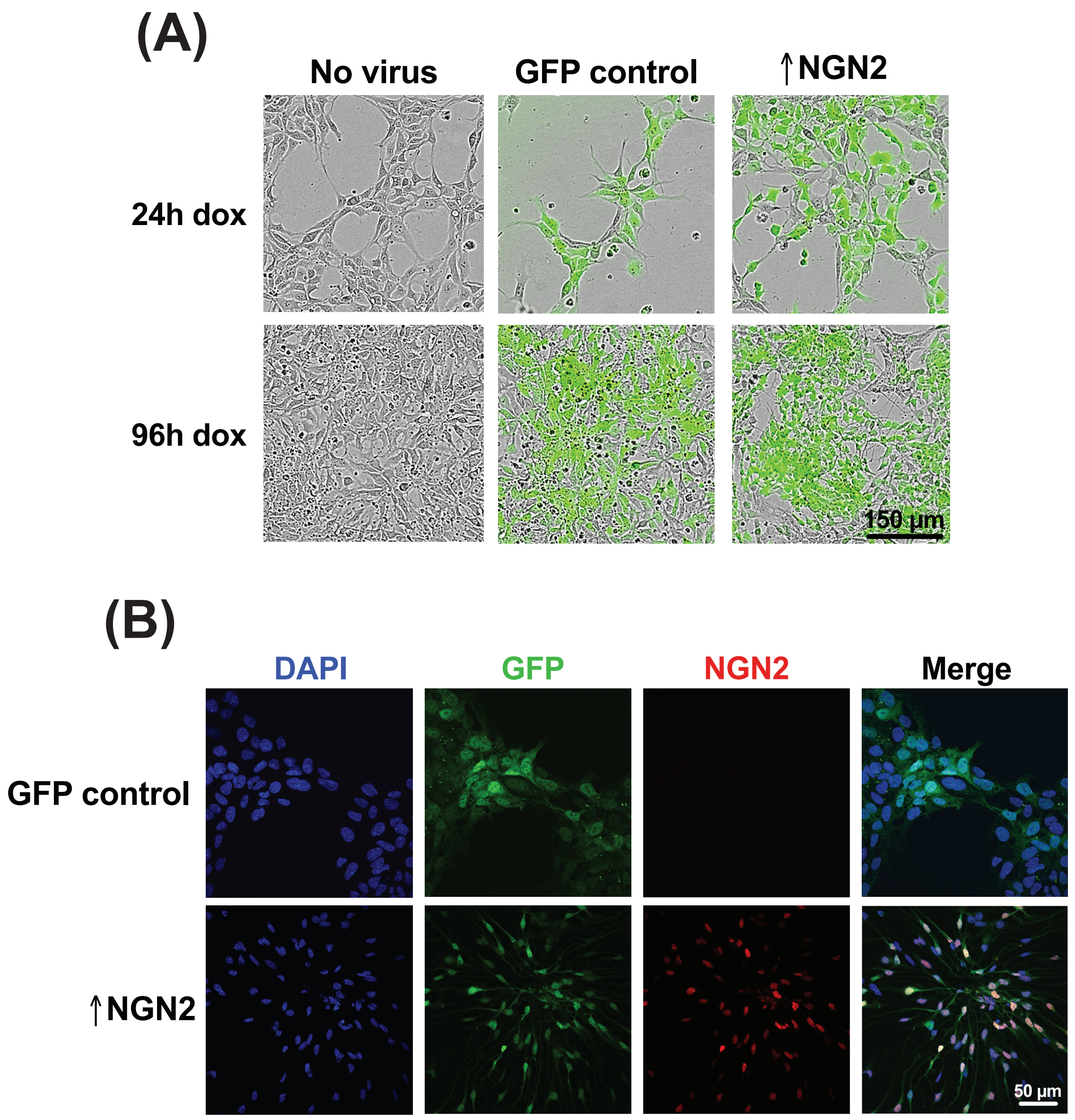

Figure S1: Confirmation of GFP and NGN2 expression in hPSCs. hPSCs were transduced with viral particles containing the reverse tetracycline transactivator and an empty GFP vector or the NGN2 overexpression vector. (A) Confirmation of GFP expression after 24 and 96 h of doxycycline (dox) administration. (B) Confirmation of NGN2 protein expression and cellular co-localisation with GFP.

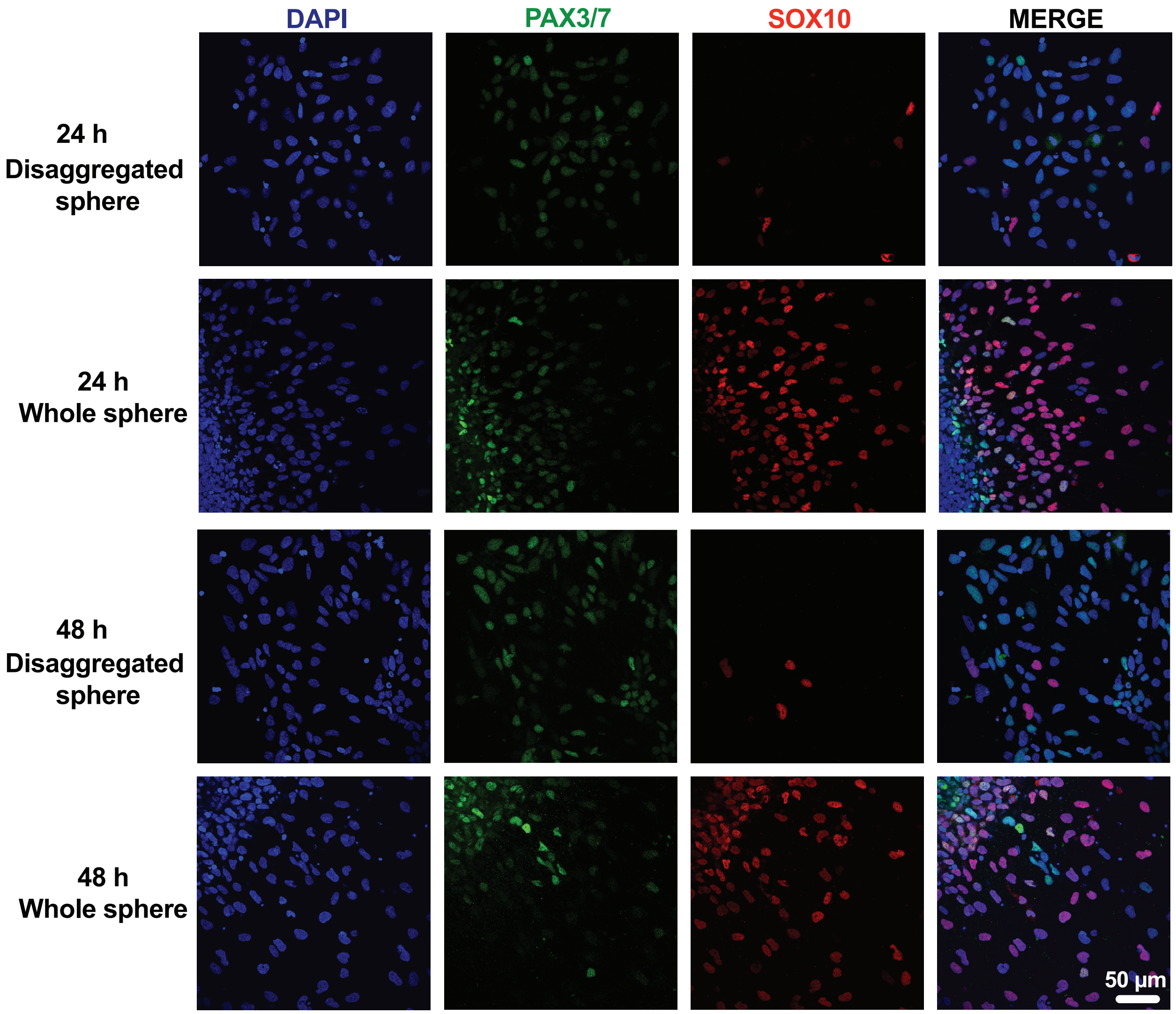

Figure S2. PAX3/7 and SOX10 expression in disaggregated and migrating neural crest cells. Representative immunocytochemistry images of the neural crest markers PAX3/7 (green) and SOX10 (red) and dapi-stained nuclei (blue).

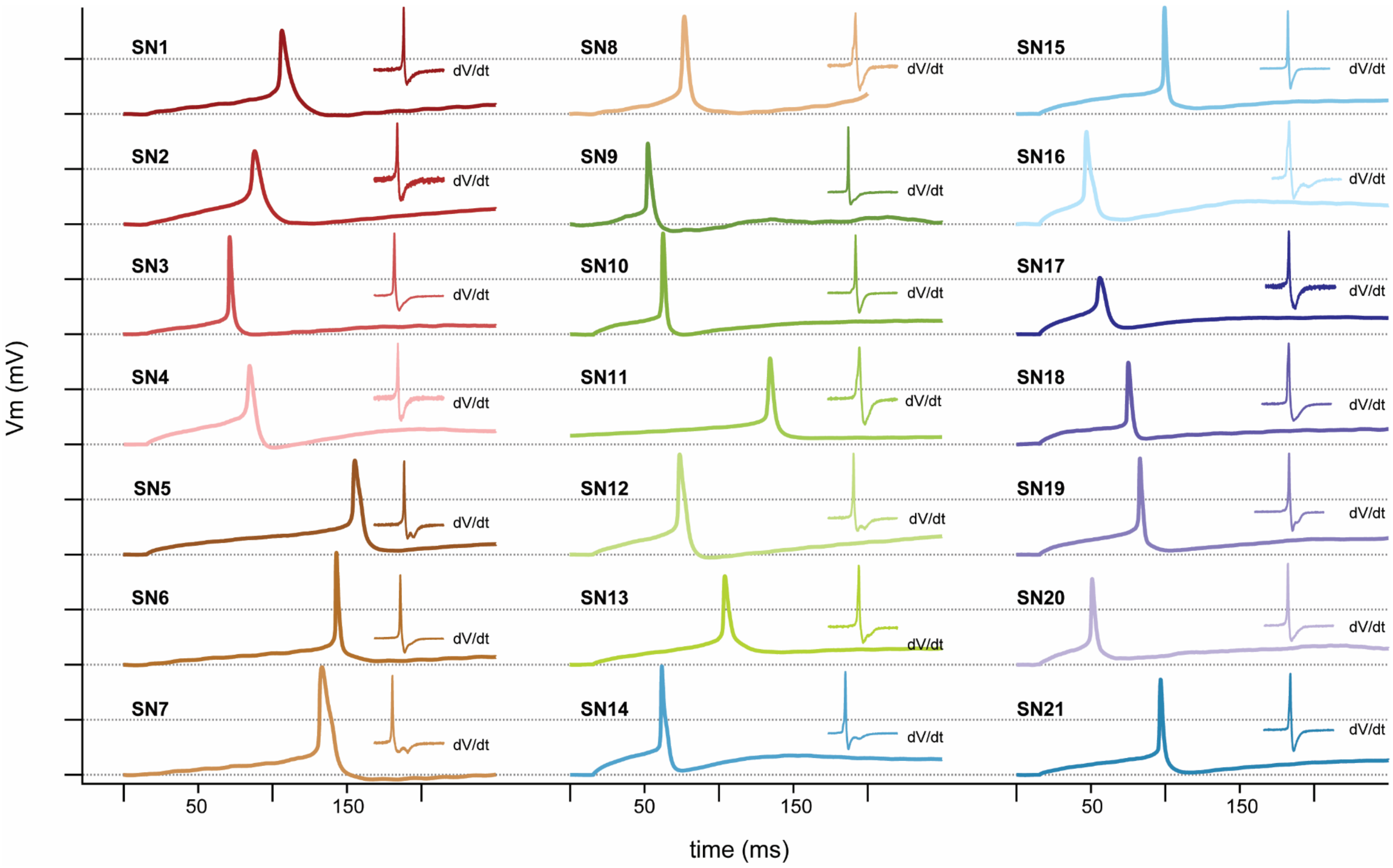

Figure S3. Action potential firing of NGN2iSN neurons at rheobase (recorded at RT ~23 °C). Insets display the corresponding first derivative dV/dt.

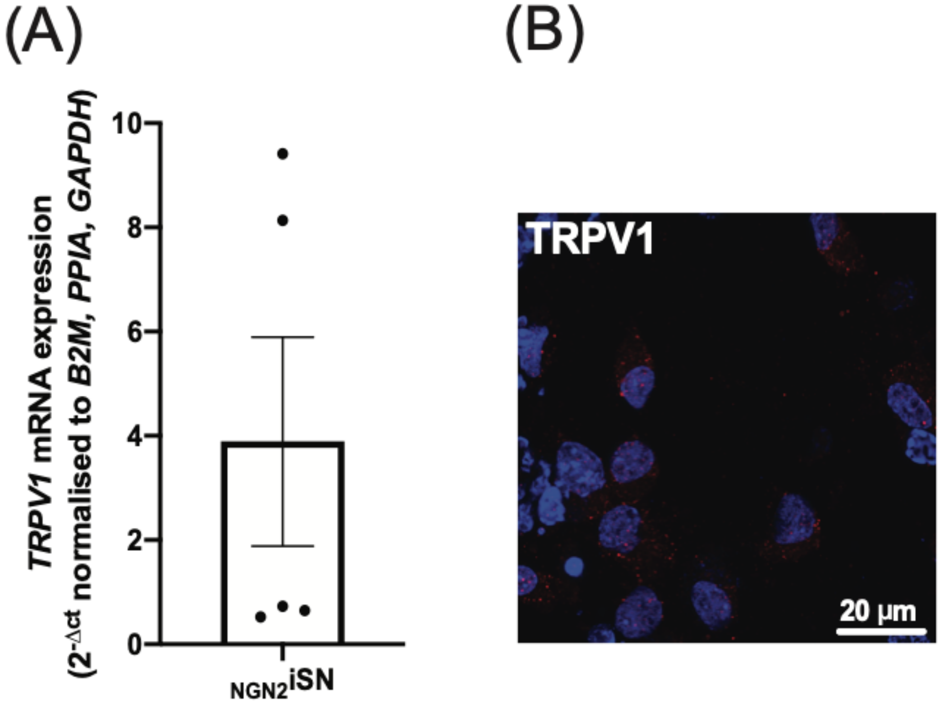

Figure S4. TRPV1 expression in NGN2iSN cultures. (A) Relative mRNA expression of TRPV1 (normalized to the house-keeping genes B2M, PPIA and GAPDH), error bars presented as SEM, n = 5 biological replicates. (B) Immunoreactivity against human TRPV1.
